# Effects of exergaming in postmenopausal women with high cardiovascular risk: A randomized controlled trial

**DOI:** 10.1002/clc.23324

**Published:** 2019-12-28

**Authors:** Eun‐Ah Jo, Shan‐Shan Wu, Hyung‐Rae Han, Jung‐Jun Park, Saejong Park, Kyoung‐Im Cho

**Affiliations:** ^1^ Convergence Medicine & Exercise Science Research Institute Kosin University College of Medicine Busan South Korea; ^2^ Division of Cardiology, Department of Internal Medicine Kosin University College of Medicine Busan South Korea; ^3^ Division of Sport Science Pusan National University Busan South Korea; ^4^ Korea Institute of Sports Science Seoul South Korea

**Keywords:** aerobic exercise, cardiorespiratory fitness, cardiovascular disease, endothelial function, epicardial fat, exergame

## Abstract

**Objective:**

Recently, exergames have been used an exercise modality as aerobic fitness activities. However, evidence of its effectiveness on cardiovascular (CV)‐related risk factors remain unclear.

**Hypothesis:**

We evaluate the effects of exergaming on CV‐related risk factors compared with traditional aerobic exercise in high CV risk patients.

**Methods:**

Sixty‐five postmenopausal women with high CV risk were randomized among exergame (n = 22), treadmill (n = 22), and control (n = 21) groups. The exergame group was engaged in the running‐based exergame using Exer Heart and the treadmill group walked or jogged on a treadmill. Cardiorespiratory fitness, flow‐mediated dilation, endothelial progenitor cells (EPCs), epicardial fat thickness, metabolic parameters, and anthropometric parameters were measured in patients before and 12 weeks after the training.

**Results:**

Exergaming significantly improved VO_2_ peak (*P* < .001; different from control, *P* < .05), flow‐mediated dilation (*P* < .001; different from control, *P* < .05), EPCs (CD34/CD117^+^, *P* < .01). Treadmill exercise was effective at improving VO_2_ peak (*P* < .01; different from control, *P* < .05), flow‐mediated dilation (*P* < .05), EPCs (CD34/CD117^+^, *P* < .01; different from control *P* < .05). Epicardial fat thickness decreased after both exercise programs (exergame, *P* < .01; treadmill, *P* < .01; no different from control).

**Conclusion:**

Exergaming showed similar effects to traditional aerobic exercise in improving cardiorespiratory fitness and endothelial function in postmenopausal women with high CV risk. These findings suggest that the exergames may serve as an alternative to conventional aerobic exercises for prevention and treatment in high CV risk patients.

## 1 INTRODUCTION

Cardiovascular (CV) disease is a major cause of premature death and morbidity worldwide. It is widely known that age, sex, hypertension, dyslipidemia, diabetes, and smoking are the main risk factors which can cause CV disease.^1^ Many other CV risk factors and their markers have been identified in recent studies. Among these, cardiorespiratory fitness (CRF) and endothelial dysfunction are strong predictors of the risk of developing CV disease.^2^, ^3^ The level of circulating endothelial progenitor cells (EPCs) has an inverse relationship with CV risk factors and it has been suggested that patients with CV disease have fewer circulating EPCs.^4^ In addition, epicardial adipose tissue is a visceral fat which accumulates in the epicardium of the heart, and has recently been considered as a new index of CV risk.^5^

It is well established that regular exercise (physical activity) is effective in preventing and treating CV risk factors by improving health‐related fitness.^6^ Despite the health benefits of regular exercise, many patients with CV risk factors often do not participate in regular exercise for reasons including lack of time, motivation, or interest.

In recent years, a new exercise program called exergame has been developed as a result of the technical advancements in gaming and virtual reality programs. Exergames are interactive video games which provide the opportunity to increase physical activity by requiring movement of the entire body and are proving to be an alternative exercise modality.^7^ In addition, exergames are used to improve senior physical performance,^8^ and facilitate poststroke motor rehabilitation.^9^ However, only a few studies have examined the effects of exergaming on CV or chronic disease‐related risk factors. Furthermore, in order for exergames to progress as a rehabilitation program for the prevention and treatment of CV disease in the future, it is necessary to compare it to traditional aerobic exercises.

Therefore, the aim of this study was to evaluate the effects of exergaming on CRF, endothelial function, epicardial fat thickness (EFT), cardio‐metabolic, and anthropometric parameters compared with treadmill exercises in high CV risk patients.

## 2 METHODS

### 2.1 Subjects

This study was a single‐site parallel randomized controlled trial (trial registration: NCT04042896). Data were collected from April 2017 to March 2018 at the Kosin University Gospel Hospital. The procedures of this study were approved by the Institutional Review Board of Kosin University Gospel Hospital. Sixty‐five female patients with a Framingham CV disease 10‐year risk score above 20% were enrolled in this study.^10^, ^11^ In order to reduce the margin of error due to sex differences and physiological responses to the greatest degree possible, only postmenopausal women were recruited considering the hormonal changes which occur during menstruation. Also, they should have no history of participating in regular exercise within the past 3 months. The exclusion criteria were chronic obstructive pulmonary disease, valvular heart disease, a history of acute coronary syndrome, a positive treadmill test, or musculoskeletal patients for whom exercise was impossible.

### 2.2 Study design

Subjects were randomized to the exergame (n = 22), treadmill (n = 22), or control (n = 21) groups. All subjects were tested over a 2‐day period before and after 12 weeks of training. On the first visit, the subject's height, weight, waist circumference (WC), blood sampling, and blood pressure (BP) were obtained after 8 hours of fasting. On the second visit, brachial artery flow‐mediated dilatation (FMD), EFT measured by echocardiography, and CRF tests were performed after 4 hours fasting. In addition, subjects were asked to refrain from excessive exercise, overeating, and caffeine consumption for 24 hours before all tests.

### 2.3 Measurement of CRF

The CRF test was carried out on a programmable treadmill (GE CASE T2100; GE Medical Systems, Milwaukee, Wisconsin) using a Ramp protocol until exhaustion.^12^ The cardiac rhythm was continuously monitored with a 12‐lead electrocardiogram system. BP North Carolina). heart rate (HR) was recorded at the end of each 3‐minute stage, at peak exercise and at 1‐minute intervals throughout recovery. Ventilation (VE), oxygen uptake (VO_2_), and carbon dioxide output (VCO_2_) were measured using a computerized system (Cosmed K4b2, Cosmed Ltd., Rome, Italy). The VE/VCO_2_ slope which relates the rate of increase in VE per unit increase in CO_2_ was calculated using the whole exercise period. All patients underwent a progressive incremental treadmill test until volitional fatigue. Peak oxygen consumption was defined as the highest VO_2_ observed during exercise test averaged for a 30 seconds recording during the last minute.

### 2.4 Measurements of circulating EPCs

For EPCs analysis, peripheral blood mononuclear cells were separated using Ficoll (Histopaque 1077; Sigma‐Aldrich, St. Louis, Missouri). Positive cells were double‐stained with anti‐CD34‐FITC (348 053; BD Pharming, San Diego, California) and anti‐KDR‐PE (FAB357P; R&D Systems), anti‐CD34‐FITC and anti‐CD117‐PE (555 714; BD Pharming), and anti‐CD34‐FITC and anti‐CD133‐PE (130‐080‐801; Miltonic Biotic, Bergisch Gladbach, Germany) monoclonal antibodies. Negative controls were also double‐stained with FITC mouse IgG1 isotype control (555 909; BD Pharmingen) and PE mouse IgG1 isotype control (349 043; BD Pharmingen) antibodies. Data analysis was performed using CellQuest Pro software (BD Biosciences). CD34^+^ KDR^+^, CD34^+^ CD117^+^, and CD34^+^ CD133^+^ double‐positive cells were defined as circulating EPCs after gating on the lymphocyte population. The total number of positive cells was calculated based on the absolute leukocyte count × percentage of positive cells (%) and expressed as the absolute number of cells per 1 mL of whole blood.^13^

### 2.5 Measurement of FMD and EFT

FMD was measured in the brachial artery according to current guidelines.^14^ Two‐dimensional ultrasonography (Vivid 7; General Electric, Horten, Norway) was performed using a 10‐MHz linear‐array transducer probe. After the baseline measurement, reactive hyperemia was induced by the inflation of a pneumatic cuff to 180 to 200 mm Hg (50 mm Hg higher than SBP) for 5 minutes on the forearm. For the peak diameter of the brachial artery, the diameter was recorded 40 to 60 seconds after sudden deflation of the cuff. The percent FMD induced by reactive hyperemia was expressed as the relative change from baseline (%FMD = 100 × [diameter after hyperemia‐baseline diameter]/baseline diameter). At the peak of the R wave of the surface electrocardiogram, each diameter was measured three times during two heartbeats and the average values were used for the final analysis.

Echocardiographic assessment of the EFT was defined as the echo‐free space between the outer wall of the myocardium and the visceral layer of the pericardium.^15^ Standard ‐dimensional echocardiography was performed with the subject in the left lateral decubitus position using a 3.5 MHz transducer (Philips iE33, Philips Medical Systems, Bothell, Washington). The echocardiographic EFT test was conducted vertically from the free wall of the right ventricle at the end‐systolic point in three heart cycles. The EFT value was recorded by considering the average of the parasternal long axis, parasternal short axis, and apical four‐chamber view. In addition, to minimize observational bias in a priori and post hoc analyses, the researcher was blinded to the baseline value.

### 2.6 Exercise training

**Figure 1:**
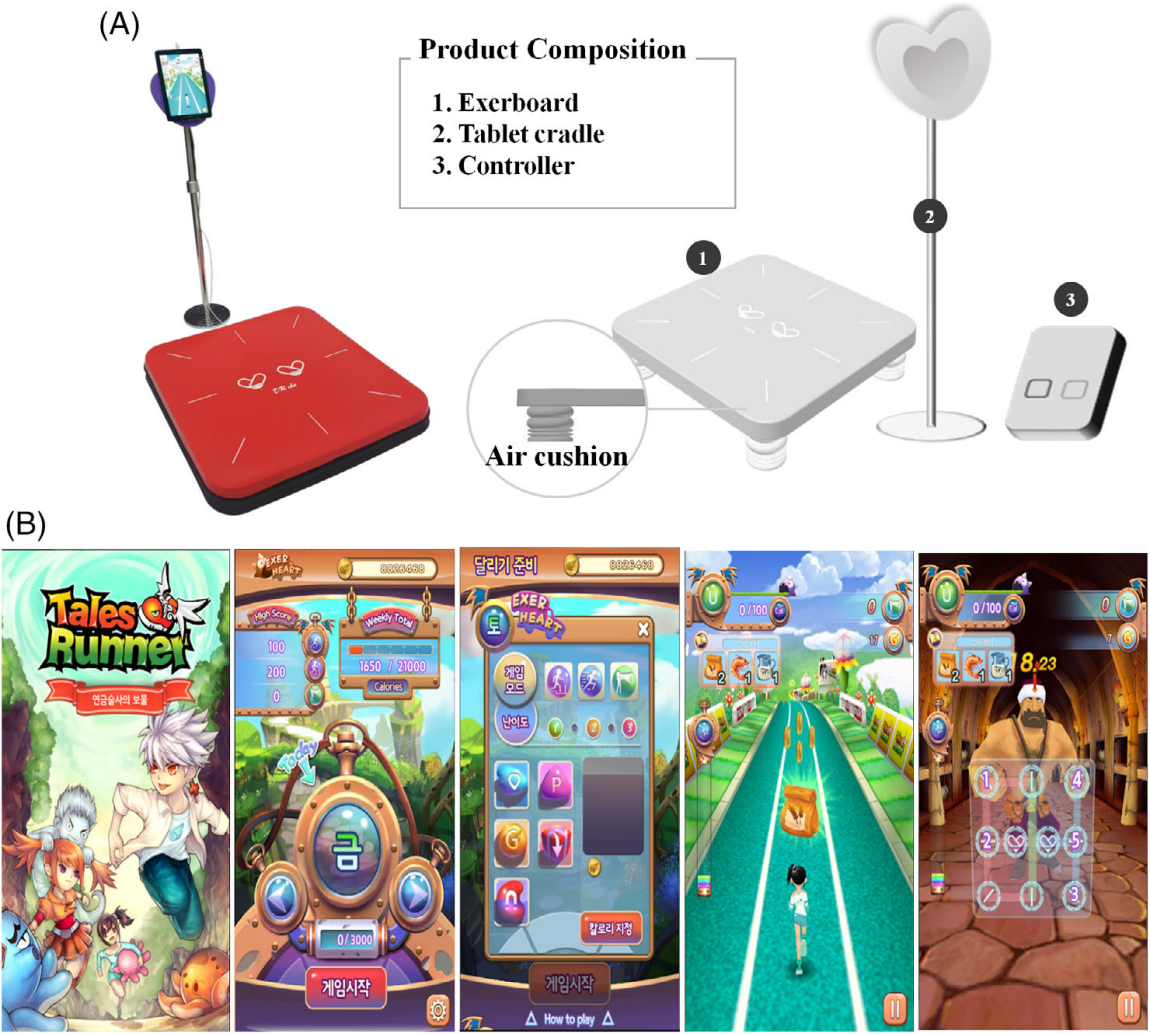
A, The exergaming group performed exercise using Exerheart devices by permission of D&J Humancare which had the copyright holder of Exerheart. B, Features of the video game “Alchemist's Treasure”

The exergame group performed exercise using the Exer Heart device (D&J Humancare, Seoul, South Korea), which consisted of a running/jumping board and a screen connected to the board (Figure 1A). The exercise program “Alchemist's Treasure,” a running‐based exergame, moves the avatar according to the user's motions and was used for the exercise session. “Alchemist's Treasure” is a game in which the user runs with the avatar, avoiding obstacles, and wins items using the front, back, left, and right sensors on the exercise board (Figure 1B).

The biggest advantage of the exergame is enjoyment. This study did not enforce exercise intensity in order to allow participants to enjoy the exergame. Thus, during the training period, the participants exercised at a self‐selected pace for 40 minutes per day. Instead, we monitored individual exercise intensity by monitoring HR (Polar RS400sd; Madison Height, Michigan) and recorded the resting, minimum, maximum, and average HR during the exergame training period. The resting HR of the subjects was 79 ± 12 beats per minute (bpm), the minimum HR was 98 ± 26 bpm, the maximum HR was 153 ± 28 bpm, and the mean HR was 120 ± 19. According to the guidelines of the American College of Sports Medicine (ACSM),^16^ the range of exercise intensity for exergaming is between 42% and 82% of HR reserve.

The treadmill group consisted of 40 minutes of walking or jogging at 60% to 80% of the HR reserve. The exercise intensity was determined using the Karvonen method target HR = [exercise intensity × (HR_max_ ‐ resting HR)] + resting HR. The HR was recorded during each session using an HR monitor (Polar RS400sd). The control group was asked to maintain their regular physical activity level for 12 weeks.

Exercise training was conducted at the Kosin University Convergence Medicine & Exercise Science Research Institute under the expert supervision of a director. For both the exergame and treadmill groups, exercise training was comprised of a 5 minutes warm‐up, a 40 minutes main exercise, and a 5 minutes cool‐down. Participants who did not perform more than 80% of the 12 weeks exercise program were excluded from this study.

### 2.7 Statistical analysis

Baseline characteristics across groups were compared using a one‐way analysis of variance (ANOVA) for continuous variables. Inter‐ and intragroup comparisons of the variables were made by two‐way ANOVA (group vs time) with repeated measurements. Bonferroni post hoc analysis was used to determine significance of data that was indicated by one‐way or two‐way ANOVA. two‐way ANOVA with repeated measures (group × time) was used to compare the data between groups. Bonferroni post hoc analysis was performed to determine significance of data that was indicated by one‐way or two‐way ANOVA. All values are presented as the mean ± SD. Statistical significance was set to *P* < .05. For statistical analysis, SPSS 21.0 (SPSS, Chicago, Illinois), a statistical program for Windows, was used.

## 3 RESULTS

**Figure 2:**
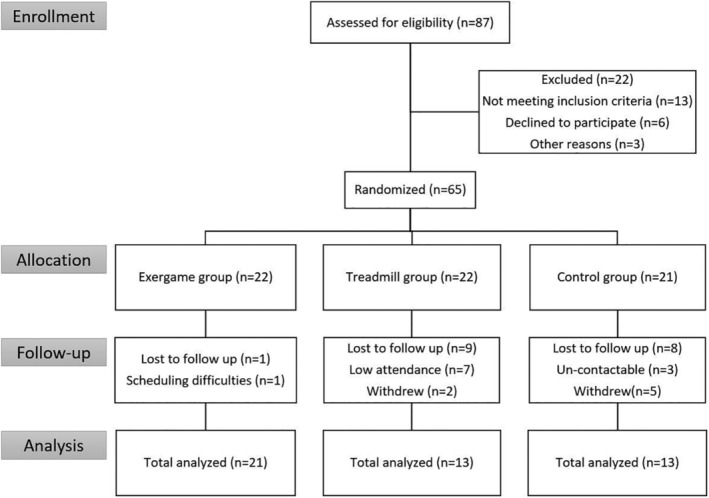
Study flow diagram

Over the course of the experimental period, 18 subjects were unable to finish the training program (Figure 2). Therefore, a total of 47 subjects completed the planned exercise training sessions. Exercise training compliance was different between the exergame (95.5%) and treadmill groups (86.1%).

There were no significant differences between continuous variable characteristics at baseline (Table 1). The WC of the subjects in the exergame and treadmill groups decreased modestly by 1.1% and 2.2%, respectively (*P* < .05 and *P* < .01, with no group differences; Table 2). Weight and body mass index (BMI) decreased modestly only in the treadmill group as well (both *P* < .05, with no group differences; Table 2). After 12 weeks of exercise intervention, the systolic BP and diastolic BP did not change significantly in any of the groups (Table 2). The lipid profiles and glucose levels did not change significantly for any group after the follow‐up period (data not shown).

**Table 1:** Baseline characteristics of participants

	Exergame (n = 22)	Treadmill (n = 22)	Control (n = 21)	*P*‐value
Risk score (%)	25.6 ± 7.0	23.9 ± 2.6	28.6 ± 12.7	.341
Age (years)	61.8 ± 10.1	57.3 ± 8.4	62.5 ± 13.9	.411
Height (cm)	154.8 ± 5.7	158.8 ± 5.2	152.9 ± 6.8	.040
Weight (kg)	66.4 ± 7.6	68.1 ± 7.5	64.0 ± 12.9	.524
BMI (kg/m^2^)	27.7 ± 3.0	27.0 ± 3.0	27.3 ± 4.6	.832
WC (cm)	94.8 ± 10.1	94.8 ± 10.1	92.1 ± 11.6	.604
Office SBP (mm Hg)	130.6 ± 17.7	135.1 ± 15.9	135.2 ± 18.3	.744
Office DBP (mm Hg)	77.1 ± 9.5	79.5 ± 9.9	75.5 ± 11.9	.572
Total cholesterol (mg/dL)	146.2 ± 26.1	144.5 ± 17.0	150.6 ± 4.8	.800
HDL cholesterol (mg/dL)	47.3 ± 11.1	54.2 ± 7.3	52.3 ± 11.5	.137
LDL cholesterol (mg/dL)	78.2 ± 25.9	77.3 ± 17.9	80.2 ± 18.2	.942
Triglyceride (mg/dL)	150.8 ± 110.9	107.7 ± 38.3	127.0 ± 49.6	.324
Fasting glucose (mg/dL)	122.5 ± 24.3	107.5 ± 17.5	113.0 ± 23.9	.160
HbA1c (%)	6.7 ± 0.9	6.1 ± 0.6	6.7 ± 0.2	.065

*Note*: Data are presented as mean ± SD.

Abbreviations: BMI, body mass index; DBP, diastolic blood pressure; HbA1c, hemoglobin A1c; HDL, high‐density lipoprotein; LDL, low‐density lipoprotein; SBP, systolic blood pressure; WC, waist circumference.

**Table 2:** Parameters before and after the experimental protocol

	Exergame (n = 21)		Treadmill (n = 13)		Control (n = 13)	
	Pre	Post	Pre	Post	Pre	Post
Weight (kg)	66.4 ± 7.6	65.7 ± 7.6	68.1 ± 7.5	67.4 ± 7.1*	64.0 ± 12.9	63.3 ± 12.8
BMI (kg/m^2)^	27.7 ± 3.0	27.4 ± 3.0	27.0 ± 3.0	26.7 ± 2.8*	27.3 ± 4.6	27.0 ± 4.7
WC (cm)	94.8 ± 10.1	93.8 ± 9.8*	91.8 ± 6.8	89.8 ± 5.5**	92.1 ± 11.6	91.3 ± 11.5
Office SBP (mm Hg)	130.6 ± 17.7	135.2 ± 11.5	135.1 ± 15.9	138.4 ± 17.2	135.2 ± 18.3	133.2 ± 19.3
Office DBP (mm Hg)	77.1 ± 9.5	77.5 ± 6.7	79.5 ± 9.9	81.2 ± 10.7	75.5 ± 11.9	76.3 ± 10.4
VE/VO_2_ slope	34.9 ± 7.7	31.8 ± 5.5*	32.8 ± 4.3	30.9 ± 5.0	31.3 ± 6.1	31.4 ± 5.7
Peak HR (bpm)	137.4 ± 15.8	144.7 ± 16.4*,^†^	134.9 ± 21.3	141.1 ± 18.2*,^†^	128.4 ± 23.4	121.9 ± 25.9
HR recovery	119.2 ± 15.8	115.5 ± 13.6	110.4 ± 18.3	114.3 ± 12.5	107.5 ± 20.9	105.0 ± 22.6
Exercise time (min)	10.8 ± 2.4	12.9 ± 2.1***,^†^	12.0 ± 2.1	13.5 ± 2.2 *,^†^	9.0 ± 3.6	9.1 ± 3.3
CD34^+^/KDR^+^ (per μL)	13.8 ± 35.0	17.1 ± 22.2	1.5 ± 3.8	19.2 ± 17.5**,^†^	6.9 ± 9.5	6.2 ± 11.9
CD34^+^/CD117^+^ (per μL)	18.1 ± 22.7	35.7 ± 40.6**	10.0 ± 12.2	16.2 ± 19.8	6.9 ± 11.1	9.2 ± 11.2
CD34^+^/CD133^+^ (per μL)	20.0 ± 48.1	12.9 ± 24.5	5.4 ± 6.6	13.1 ± 14.4	3.1 ± 4.8	5.4 ± 9.7

*Note*: Data are presented as mean ± SD. Different from before and after at same group (*P < 0.05; **P < 0.01; ***P < 0.001); different from control at the same period (^†^ *P* < 0.05).

Abbreviations: BMI, body mass index; DBP, diastolic blood pressure; EFT, epicardial fat thickness; FMD, flow‐mediated dilation; HR, heart rate; SBP, systolic blood pressure; VO_2_ peak, maximal oxygen uptake; WC, waist circumference.

**Figure 3:**
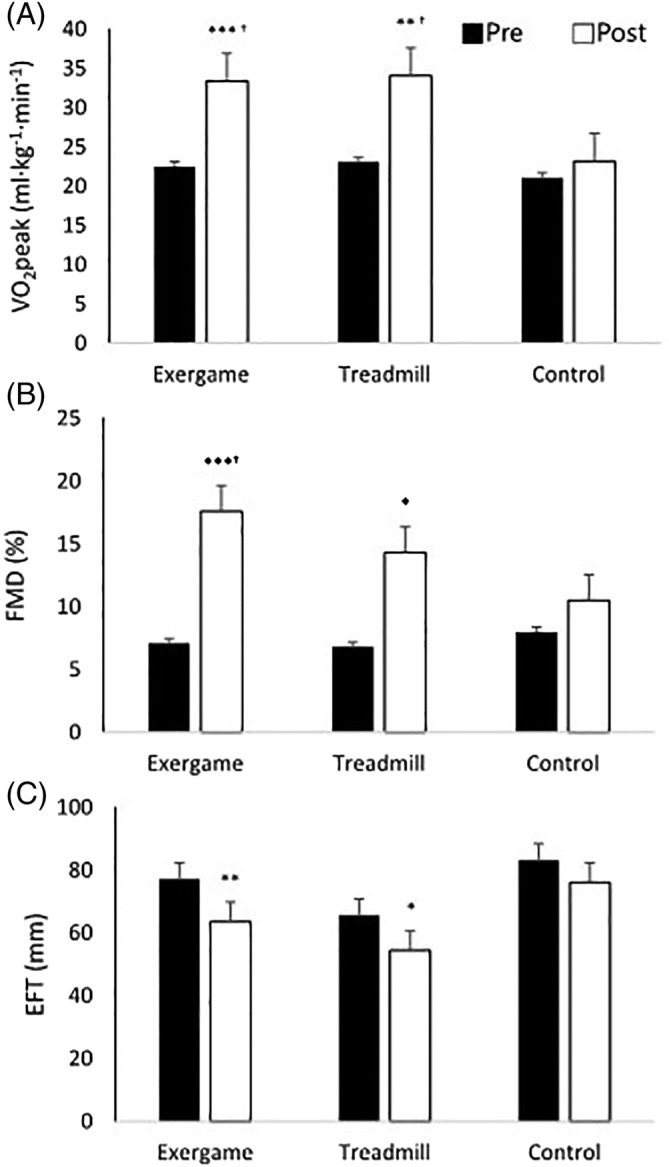
Changes in cardiorespiratory fitness (VO_2_ peak, A), endothelial function measured as flow mediated dilation (FMD, B) and epicardial fat thickness (EFT, C) before (pre) and after 12 weeks training program (post). Different from before and after at same group (*P < .05; **P < .01; ***P < .001); different from control at the same period (^†^ *P* < .05)

The baseline VO_2_ peak, VE/VCO_2_ slop, peak HR, HR recovery, and exercise time values were similar across groups. After the 12 weeks training period, the VO_2_ peak increased by 48% and 47%, respectively, in the exergame and treadmill groups (*P* < .001 and *P* < .01, difference from control, both *P* < .05, Figure 3A). A small, significant decrease in VE/VCO_2_ slop was shown in exergame group (*P* < .05; no difference from control). The peak HR significantly increased by 5.3% and 4.6%, respectively, in the exergame and treadmill groups (*P* < .001 and *P* < .05, difference from control, both *P* < .05; Table 2), but there was no change in HR recovery. In addition, the exercise time significantly increased by 19% and 13% in the exergame and treadmill groups, respectively (*P* < .001 and *P* < .05, difference from control, both *P* < .05; Table 2). There were no differences between exergame and treadmill group for VO_2_ peak, peak HR, and exercise time.

Endothelial function (FMD and EPCs) at the baseline was similar between groups. The FMD for the exergame and treadmill groups improved by 10.5% (*P* < .001, difference from control, *P* < .05) and 7.5% (*P* < .05, no difference from control), respectively (Figure 3B). In this study, we analyzed CD34/KDR^+^ cells, which are typical markers of EPCs, and CD34/CD133^+^ and CD34/CD117^+^ cells, which are known to be involved in neovascularization.^17^ After the 12 weeks training period, the number of CD34/KDR^+^ cells significantly increased in the treadmill group (*P* < .01, difference from control, *P* < .05) and there was no change in the exergame group (Table 2). In contrast, the number of CD34/CD117^+^ cells significantly increased in the exergame group (*P* < .01, no difference from control) and did not change in the treadmill group (Table 2). There was also no significant difference between the two groups. There were also no differences between exergame and treadmill group for FMD and EPCs.

The EFT at baseline was similar between the groups. Although there was no significant difference between the groups, significant decreases of 18% in the exergame group and 17% in the treadmill group were observed after12 weeks (*P* < .01 and *P* < .05; Figure 3C).

## 4 DISCUSSION

To the best of our knowledge, our study is the first to compare the effects of exergames and traditional treadmill exercise in risk factors associated with CV disease in high CV risk patients. Our results indicate that (a) a running‐based exergame with Exer Heart, when played at self‐selected intensity, similarly improved CRF and endothelial function in high CV risk patients compared to the traditional treadmill exercise performed at a moderate intensity, (b) a running‐based exergame with Exer Heart showing potential for use as CV fitness tool, which can be an alternative exercise modality as effective as traditional treadmill exercise, and (c) enjoyment factors in exergame can promote attendance or continued adherence.

Of all established risk factors, low aerobic exercise capacity appears to be the strongest predictor of mortality.^2^ The ACSM states that an intensity of 40%to 85% or 50% to 85% of HR reserve is needed for adults to achieve improvements in CV fitness while those with low fitness can still experience improvements with lower intensities around 40% to 49% HR reserve.^16^ In this study, the reason why the running‐based exergame, played at self‐selected intensity, experienced CRF improvements may be that the intensity of “Alchemist's Treasure” with Exer Heart was satisfied the minimum exercise intensity requirement of the ACSM for CRF improvement.

However, there are many interpretations for the advantages of exergames based on currently available research results. According to some studies, exergames can induce CV responses which are similar or superior to traditional, mid‐high intensity physical activities, which are within the recommended ranges of the ACSM for CRF training.^18^, ^19^ In contrast, according to some studies, game systems cannot provide energy expenditures similar to those obtained from mid‐high intensity physical activity and fitness advantages.^7^, ^20^ Although exergaming did not change metabolic parameters in this study, our study showed that CRF in high CV risk patients can be improved without any drastic improvements in lipid profiles or glucose levels. However, due to the inconsistent results obtained in many studies to date, there is a need for future research on additional subject groups. Exercise intensity can be held constant in treadmill exercises using speed and inclination angles. Whereas comparatively variable complex activities are performed in exergaming, recommendations on the exercise type (continuous, interval, etc.), intensity, and energy expenditure are required, and the goal of the exercise must be defined for each subject based on those results.

The benefits of traditional exercise to improve endothelial function in CV or chronic disease patients have been reported numerous times. However, studies on the effect of exergame on endothelial function is limited. In addition, this study is a pioneering study in the evaluation of the effects of exergame on EPCs in high CV risk patients. Although both exergame and treadmill improved FMD and EPCs in this study, FMD showed improvement in exergame group and EPCs in treadmill group compared with control group. Since both FMD and EPCs were representative markers of endothelial function, it is thought that the two exercise training types were effective in improving endothelial function.

Increase in nitric oxide (NO) production leads to vasodilation of arterioles, and endothelial NO synthase (eNOS) and vascular endothelial growth factor (VEGF) seem to play a key role in the increased mobilization of EPCs.^21^ Exercise training promotes higher VO_2_ and blood flow in skeletal muscle, leading to an increase in hypoxia and shear stress, respectively, subsequently causing elevated NO, eNOS, and VEGF production.^21^ Although we did not measure factors, such as NO, eNOS, and VEGF levels which may be correlated with FMD and EPCs mechanisms, both exergame and treadmill exercises programs induced shear stress on the vascular wall and seemed to increase NO bioavailability, improve circulating EPCs levels, and improve overall endothelial function.

In this study, although there was no difference from the control group, 12 weeks of exergame training reduces WC 1.1% and EFT 17.6%, and treadmill training decreases BMI 1.1%, WC 2.2%, and EFT 17.2%. Kim et al.^22^ reported that the EFT of obese men decreased by 8.61% after 12 weeks of exercise training, and that the BMI and WC decreased by 4.3% and 4.2%, respectively. The degree of EFT reduction effect was higher in our study compared to a previous study conducted by Kim et al., but the decrease in BMI and WC were smaller in our study. This is probably because subjects in this study were mostly overweight CV high‐risk patients rather than obese.

Some of the possible mechanisms proposed for the reduction of visceral adipose tissue include the secretion of fat‐dissolving hormones, the increase of energy consumption after exercise, and the oxidation of fats which favor greater negative energy balance.^23^ Although the mechanisms involved in epicardial adipose tissue are not well‐known, epicardial adipose tissue is an indicator of visceral adipose tissue.^24^ Therefore, we can infer that the same mechanism may have resulted in an EFT reduction in this study. Another mechanism is the high correlations between inflammation markers and EFT.^25^ And Many studies have reported that exercise training reduces inflammatory markers throughout the body.^26^

In this study, above all, exergame group showed higher attendance and adherence than the treadmill group. Although the enjoyment scale was not measured in this study, it was considered that exergaming provided better enjoyment and motivation to patients than treadmill exercise. Some studies show that exergaming produced the greatest level of enjoyment compared with traditional exercise activities.^27^, ^28^ Enjoyment in an activity can promote attendance or continued adherence.^29^ High attendance and adherence increased amount of physical activity, which leads to improved health benefits.

We do acknowledge limitations to the study that may have impacted our outcomes. This study only tested the “Alchemist's Treasure” game with the Exer Heart program. Therefore, the results of our study cannot be generalized to all exergame exercise programs. Also, only female patients were recruited to reduce the margin of error in the data due to sex differences. Future studies involving male patients are also needed. Finally, there was low adherence and high drop‐out in treadmill and control group. On the other hand, that reflect realistic issues, because it is not easy for patients who had no exercise experience to continue the traditional aerobic treadmill exercise. We realized that enjoyment and motivation in exercise are important, and we emphasized that. Future studies comparing enjoyment scale of exercise types are needed.

## 5 CONCLUSION

This study showed that exergame played at a self‐selected intensity showed identical effects as vigorous‐intensity traditional exercise on a treadmill through changes in CFR, endothelial function, and epicardial fat distribution in high CV risk patients. Moreover, the exergame group showed higher attendance and adherence than the treadmill group. Based on these results, it may be concluded that exergaming program satisfies the recommendations of the ACSM for physical activity and may represent an alternative to traditional treadmill exercises. Together, the enjoyment and comparatively high motivation provided by exergaming provide support for its potential as an attractive exercise modality for the prevention and treatment of CV risk.

## CONFLICT OF INTEREST

The authors declare no potential conflict of interests.
